# High rate of reoperation and conversion to total hip arthroplasty after internal fixation of young femoral neck fractures: a population-based study of 796 patients

**DOI:** 10.1080/17453674.2018.1558380

**Published:** 2019-02-04

**Authors:** David J Stockton, Lyndsay M O’Hara, Nathan N O’Hara, Kelly A Lefaivre, Peter J O’Brien, Gerard P Slobogean

**Affiliations:** a Department of Orthopaedics and Clinician Investigator Program, University of British Columbia, Vancouver, British Columbia, Canada;; b Department of Epidemiology & Public Health, University of Maryland, Baltimore, MD, USA;; c Department of Orthopaedics, University of Maryland, R Adams Cowley Shock Trauma Center, Baltimore, MD, USA

## Abstract

Background and purpose — Most often, the goal of non-geriatric femoral neck fracture surgery is to preserve the native hip joint. However, reoperations for painful implants, osteonecrosis, and nonunion are common. We determined the reoperation rate and time-to-reoperation following internal fixation of these fractures in a large population cohort.

Patients and methods — This retrospective cohort study included patients between the ages of 18 and 50 years old who underwent internal fixation for a femoral neck fracture during 1997–2013. Patients were followed until December 2013. Primary outcomes were reoperation rate and time-to-reoperation. Time-to-event analysis was performed to estimate the rate of any reoperation and for THA specifically, while testing the dependency of time-to-reoperation on secondary variables.

Results — 796 young femoral neck fracture patients were treated with internal fixation during the study period (median age 43 years, 39% women). Median follow-up was 8 years (IQR 4–13). One-third underwent at least 1 reoperation at a median 16 months after the index surgery (IQR 8–31). Half of reoperations were for implant removal, followed by conversion to total hip arthroplasty. 14% of the cohort were converted to THA. The median time to conversion was 2 years (IQR 1–4). Neither female sex nor older age had a statistically significant effect on time-to-reoperation or time-to-THA conversion.

Interpretation — Following internal fixation of young femoral neck fracture, 1 in 3 patients required a reoperation, and 1 in 7 were converted to THA. These data should be considered by patients and surgeons during treatment decision-making.

Femoral neck fractures in non-geriatric adults are a challenge to treat successfully. They often occur from high-energy trauma and result in displaced fracture patterns (Protzman and Burkhalter 1976, Robinson et al. 1995). Reduction and internal fixation is performed for nearly all younger patients with these fractures in order to preserve the native hip joint (Tooke and Favero 1985, Ly et al. 2008). The risk of a healing complication is often high, with the most common causes being osteonecrosis (14%), nonunion (9%), and severe femoral neck shortening (13–32%) (Slobogean et al. 2015, Stockton et al. 2015, Slobogean et al. 2017). A recent meta-analysis estimated a reoperation rate of 18% following internal fixation of young femoral neck fractures; however, this rate was estimated using predominantly retrospective, short-term, case series data (Slobogean et al. 2015). Lin et al. (2014) estimated the 10-year complication-free rate for femoral neck fractures in patients age 20–40 years to be 67% in Taiwan, but the outcome was an aggregate of reoperations and medical complications. While several case series have described the short-term surgical complications of these injuries, long-term population-based studies remain lacking; it is unknown how many patients treated with internal fixation will eventually require a reoperation or conversion to total hip arthroplasty (THA).

Controversy exists surrounding the role of arthroplasty in young femoral neck fractures. In these patients, THA has historically been foregone as a primary intervention due to the finite lifespan of the implant. THA is typically considered a secondary procedure for the treatment of complications, such as osteonecrosis or nonunion (Angelini et al. 2009, Pauyo et al. 2014). However, the use of THA as a secondary procedure for internal fixation is associated with a higher complication rate (McKinley et al. 2002, Mahmoud et al. 2016). Advancements in arthroplasty systems have demonstrated improved survivorship, increasing their viability as a primary treatment for younger patients (Adelani et al. 2013). A better understanding of current rates of complications and THA conversions for younger patients with femoral neck fractures is imperative to guide the treatment choice between internal fixation and primary THA, particularly for middle-aged patients.

Therefore we determined the reoperation rate (in total and specifically for conversion to THA) and time-to-reoperation for patients aged 18–50 after internal fixation of femoral neck fractures. Secondarily, we sought to determine the relationship between age, sex, and hospital volume on the rates of reoperation and conversion to THA in this patient population. We hypothesized that male sex, older age, and having the index procedure done at a low-volume center would be associated with an increased risk of reoperation.

## Patients and methods

This was a retrospective cohort study. We used British Columbia (BC) administrative health-care data collected and linked by Population Data BC, a multi-hospital data and education resource facilitating interdisciplinary research on the determinants of human health, well-being, and development of the citizens of BC. The main data sources were Medical Services Plan (MSP) Payment Information Files that capture data on medically necessary services provided by physicians (i.e., licensed orthopedic surgeons) to individuals covered by MSP, the province’s universal insurance program (British Columbia Ministry of Health 2016a); the Discharge Abstracts Database (DAD), which contains demographic, administrative, and clinical data for all patients discharged from acute-care hospitals in BC (Canadian Institute for Health Information 2016); and Consolidation Files that contain basic demographic information including geo-coding that indicates location of residence (British Columbia Ministry of Health 2016b). These databases, which provide information from 1985 onwards, are held securely in linked, de-identified form at Population Data BC (www.popdata.bc.ca).

We included all patients aged 18–50 from 1997 to 2013 who sustained femoral neck fractures (AO/OTA Type 31B) with subsequent osteosynthesis (MSP code 55751 for closed reduction internal fixation [CRIF] and 55755 for open reduction internal fixation [ORIF]) (Province of BC 2018). Patients who concomitantly experienced femoral shaft fractures (MSP codes 55782, 55783, and 55785) were also included; however, those that had pelvic or acetabular fracture (MSP code 55741, 55745, or 55746) were excluded due to the increased risk conferred to the viability of the native hip joint. Prior to delivering the final cohort for analysis, the Data Stewards at Population Data BC also excluded patients if they died or if they moved out of province after their index surgery (identified by the Province of Patient code from the DAD). All patients were followed until December 31, 2013.

Age and sex variables were also obtained from the DAD. Age was analyzed as a continuous variable and as a dichotomized categorical variable, with an age of 45 years old chosen as the separating value. 45 years old is the lowest suggested age that has been recommended in the literature for consideration of treatment by primary arthroplasty (Swart et al. 2017). Index treatment was provided at 54 unique hospitals, with each hospital treating between 1 and 59 patients during the study period. The 3 hospitals that treated more than 40 index fractures during the study period were coded as “high-volume hospitals.”

### Study outcomes

The primary outcomes were rate of reoperation and rate of conversion to THA. Rate was estimated using person-years as the denominator, such that patients with an index surgery early in the study period would contribute proportionally more to the denominator relative to those patients with more recent index surgeries (Szklo and Nieto 2014). Reoperation was defined as any of any of the following procedures undertaken after the index surgery: implant removal (MSP code 55415 or 55420), proximal femur osteotomy (55603), bone grafting (MSP code 55651), non-union fixation (MSP code 55633), hip hemi-arthroplasty (55662), and THA (MSP code 55663). Secondary outcomes included time-to-any-reoperation and time-to-THA.

### Statistics

The Pearson chi-square test was used to compare patient demographic and hospital variables by outcome status. A descriptive analysis of reoperation type was also conducted. A Kaplan–Meier analysis was performed to estimate the rate of failure of the index procedure for any reoperation and for THA specifically at 1-, 2-, 5-, and 10- year intervals as well as a mean time-to-reoperation. A Cox proportional-hazards regression model was used to study the dependency of time-to-reoperation and time-to-THA on patient and hospital variables. All statistical analyses were conducted using SAS 9.4 (SAS Institute, Cary, NC, USA) and SPSS, Version 22 (IBM Corp, Armonk, NY, USA).

### Ethics, funding, and potential conflicts of interest

The study was approved by the Clinical Research Ethics Board at the University of British Columbia (H14-03413). No external sources of funding were utilized. The fees for accessing the administrative databases held by Population Data BC were waived via a Student Waiver. None of the authors have any conflicts of interest. All inferences, opinions, and conclusions drawn in this study are those of the authors, and do not reflect the opinions or policies of the Data Stewards at Population Data BC.

## Results

The final cohort consisted of 796 patients aged 18–50 years who underwent internal fixation for femoral neck fracture between 1997 and 2013. The number of included patients ranged between 40 and 60 cases per year. The median age of the cohort was 43 years (IQR 35–48) and 61% were male (Table 1). Median follow-up time was 8 years (IQR 4–13).

**Table 1.** Demographic data (n = 796)

Variable	Reoperation	No reoperation	p-value
No. of index cases	235	561	NA
Age, mean (SD)	40 (8)	41 (9)	0.7
Male sex, n (%)	138 (59)	346 (62)	0.4
Follow-up years, mean (SD)	9 (5)	8 (5)	< 0.01
Hospital volume, n (%) ^a^			
< 40 cases	187 (80)	451 (80)	0.9
≥ 40 cases	48 (20)	110 (20)	

**^a^**Total number of young femoral neck fracture fixation cases performed from 1997 to 2013.

There were 351 reoperations performed during the study period among 235 unique patients (30%) who required at least 1 reoperation. The most common reoperation was implant removal (n = 192, 55%), followed by conversion to THA (n = 102, 29%). 52 patients (7%) in the cohort required 2 or more reoperations within the study period. Among these 52 patients, the median number of reoperations was 2 (IQR 2–4) (Table 2).

**Table 2.** Types of reoperation (n = 351 ^a^)

Type of reoperation	n (%)
Implant removal	192 (55)
Total hip arthroplasty	102 (29)
Nonunion fixation	18 (5)
Revision CRIF and/ORIF	18 (5)
Hip hemiarthroplasty	9 (3)
Bone grafting	9 (3)
Osteotomy	3 (1)

**^a^** This total includes all reoperations, not just the first reoperation.

The median time to the first reoperation was 16 months (IQR 8–31). The Kaplan–Meier survival curve (Figure) shows that the 1-, 2-, 5-, and 10-year reoperation rates were 12%, 21%, 30%, and 34% respectively. Reoperation rates were similar whether stratified by age or hospital volume (Table 3). Our results remained statistically insignificant whether age was analyzed as a continuous or categorical variable. For conversion to THA specifically, the 1-, 2-, 5-, and 10-year reoperation rates were 3%, 6%, 10%, and 14% respectively. For those patients that required conversion to THA, the median time-to-THA was 27 months (IQR 12–50).

**Table 3.** Cox proportional hazards model for risk (HR) and 95% confidence interval (CI) of reoperation and risk of THA

Variable	HR (CI)	p-value
Risk for reoperation:		
Age (45–50 years)	0.8 (0.6–1.1)	0.1
Female sex	1.2 (0.9–1.5)	0.3
Index surgery at high-volume center	0.9 (0.7–1.3)	0.6
Risk for conversion to THA:		
Age (45–50 years)	1.2 (0.8–1.9)	0.3
Female sex	1.2 (0.8–1.8)	0.5
Index surgery at high-volume center	0.7 (0.4–1.2)	0.2

Data on comorbidities, socioeconomic status, and time-to-fixation were incomplete and were not included in the multivariable model. In the final model, neither age, sex, nor hospital volume was associated with a higher risk of reoperation, or with a higher risk of conversion to THA (Table 3).

## Discussion

This study used population-based health data to estimate the long-term outcomes of young femoral neck fractures. This represents the largest North American cohort of such fractures with long-term outcomes to date. A 10-year reoperation rate of 34% and a 10-year rate of conversion to THA of 14% suggests a substantial opportunity to improve the treatment of femoral neck fractures in the non-geriatric population.

Our results are comparable to previous literature, which predominantly consists of single-center case series. The largest series, by Huang et al. (2010), followed 146 patients for at least 2 years post-surgery (mean 5 years). Overall, 33 patients (23%) were converted to a prosthetic replacement, most due to osteonecrosis. Duckworth et al. (2011) followed a series of 122 patients with displaced young femoral neck fractures and found that 29% required a reoperation and 22% conversion to arthroplasty at a mean of 11 months (0.5–39). Haidukewych et al. (2004) found an 18% rate of conversion to arthroplasty at a mean of 7 years (3 months–15 years). Using population-based, linked health data, our long-term reoperation rate was higher than in previous reports; however, our rate of conversion to THA was lower than many studies, even those with shorter follow-up. While the reason for this discrepancy is unclear, it is important to acknowledge that most case series focus on displaced femoral neck fractures whereas our population-based cohort included patients with less severe fracture patterns.

Scant population-based data exist for young femoral neck fractures. Samuel et al. (2016) performed a retrospective cohort study of 1,361 patients using the National Trauma Data Bank. They advocated for expedited care of these fractures after finding that delayed treatment (after 24 hours) resulted in higher odds of in-hospital complications. Long-term outcomes, however, were not examined. Lin et al. (2014) conducted a population-based study in Taiwan using their National Health Insurance Research Database. Of 2,905 femoral neck fractures in patients aged 20–40 years in their cohort, 67% were complication-free at 10 years. The 1-, 2-, 5-, and 10-year complication rates are similar to our reported findings at 13%, 20%, 25%, and 27%. However, direct comparisons with Lin et al. are limited given their use of a composite endpoint that included death, readmission within 90 days of index surgery, and reoperation along with their exclusion of study participants older than 40 years of age. Similarly, they did not observe an association between sex and the time to the first complication. Our findings expand on the previous literature by contributing an estimate of the median time-to-reoperation (16 months).

To our knowledge, no other study has quantified the trajectory of reoperations for young femoral neck fractures. With a median time-to-reoperation of 16 months and the initially steep Kaplan–Meier survival curve seen in Figure 1, it is evident that most reoperations occur within the first 5 years of the index surgery.

**Figure:**
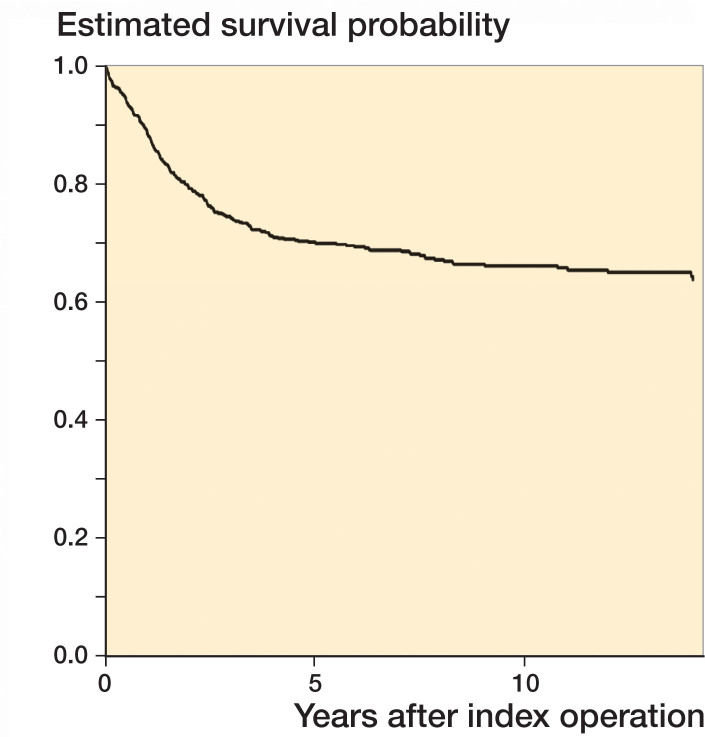
Kaplan–Meier survival curve for reoperation.

In our cohort, the most common type of reoperation was implant removal (55%) followed by THA (29%), nonunion fixation (5%), and revision internal fixation (5%). Previous studies have suggested that the most common reasons for reoperation following internal fixation of young femoral neck fracture are osteonecrosis and nonunion (Haidukewych et al. 2004, Pauyo et al. 2014); however, our results indicate that implant removal accounts for most reoperations. Implant removal does often precede more extensive operations such as THA. While the Medical Services Plan billing code for the major operation is reliably coded, the code for the more minor procedure accompanying it is not. While this represents an unfortunate reality of population-level databases, it does not account for all cases of implant removal. The fact remains that a large number of reoperations were for implant removal in isolation, which may be consistent with the issue of femoral neck shortening suspected to be associated with either an altered abductor moment arm or symptomatic implants (Slobogean et al. 2017).

Implant removal notwithstanding, hip arthroplasty (THA and hemiarthroplasty) accounted for 32% of reoperations while hip-preserving procedures (nonunion fixation, revision fixation, bone grafting, and osteotomy) accounted for 14%. An arthroplasty procedure typically denotes an unsalvageable joint, most likely due to advanced osteonecrosis or nonunion. With regard to nonunion, some surgeons in British Columbia may treat difficult cases with arthroplasty; however, in many cases the first option for an aseptic nonunion is a hip-preserving procedure. Revision fixation using a valgus-producing trochanteric osteotomy is a recommended treatment for nonunion in high Pauwels angle fractures (Deakin et al. 2015). Our results suggest that this technique may actually be quite rare (0.8%); however, we suspect that surgeons tended to bill this procedure as a “nonunion fixation” instead of an “osteotomy.” Nonunion after fracture of the young femoral neck is unique in that the treatment may involve an osteotomy, a fact that the surgical billing codes do not account for. In this instance, it is likely that surgeons chose to bill using a code that represented the indication for the procedure.

Recent evidence supporting primary total arthroplasty for difficult periarticular fractures with high complication rates has emerged for certain proximal humerus, distal femur, and elbow fractures (McKee et al. 2009, Chen et al. 2017, Sebastia-Forcada et al. 2017). There is increasing controversy over exactly what patient- and fracture-related characteristics should lead the treating surgeon to consider THA as the primary treatment for young femoral neck fractures. Converting a failed fixation to THA represents an additional burden to the individual and the health system, and does not fully restore function (Zielinski et al. 2014). In a Markov economic decision analysis, primary THA was found to be the most cost-effective intervention for young femoral neck fracture for healthy patients > 54 years old, > 47 years old for those with mild comorbidities, and > 44 years old for those with multiple comorbidities (Swart et al. 2017). That analysis used an estimated rate for failed ORIF converted to THA for patients < 50 years old of 13%, drawn from a systematic literature review. Our observed 14% arthroplasty conversion rate supports their model parameters and may even lower the transition ages at which THA becomes the more cost-effective primary intervention. However, our analysis did not find that patients age 45–50 years old had a higher probability of needing a reoperation. Discerning the appropriate primary intervention for these fractures will likely take into account multiple factors that remain to be clearly defined.

Our secondary analyses were unable to support our hypothesized associations between key variables and the risk of reoperation or conversion to THA. Neither age, sex, nor hospital volume were associated with these outcomes.

The most significant limitation of this large administrative database study is our inability to measure the fracture severity or fixation quality. Additionally, the socioeconomic, provider volume, and time-to-initial fixation data were incomplete and therefore had to be excluded from the multivariable analyses. Excluding patients that died or moved out of province may have slightly biased our estimates if these patients were more prone to reoperation. Furthermore, while our analyses focused on the important outcome of reoperation, we are unable to comment on the functional outcome of the cohort, which could be associated with hospital or patient variables. Regardless, these limitations are mitigated by the unique strength of using a linked, population-based database from a single-payer health system, which ensures that reoperations that occurred several years after the index fixation or at another hospital were accurately captured.

In summary, our study expands the understanding of young femoral neck fracture outcomes by estimating short-, medium-, and long-term reoperation rates. 1 in 3 patients will require a reoperation, while 1 in 7 will be converted to THA. These values are useful prognostic information for both surgeons and patients, and they provide further impetus for research to determine what fracture- and patient-related characteristics should be used to discern the optimal treatment for this difficult fracture.

DJS: study design, data collection, data analysis, writing of the draft paper, and revision of the paper. LMO, NNO, GPS: study design, data analysis, and revision of the paper. KAF, PJO: study design and revision of the paper. 

*Acta* thanks Hans Berg and Ville Mattila for help with peer review of this study.
